# Hemp: A Sustainable Plant with High Industrial Value in Food Processing

**DOI:** 10.3390/foods12030651

**Published:** 2023-02-02

**Authors:** Hiroyuki Yano, Wei Fu

**Affiliations:** Institute of Food Research, National Agriculture and Food Research Organization, Ibaraki 305-8642, Japan

**Keywords:** disulfide, food processing, gluten free, hemp, high protein food, sustainability, meat analogue

## Abstract

In the era of SDGs, useful plants which provide valuable industrial outputs and at the same time pose less impact on the environment should be explored. Hemp seems one of the most relevant gluten-free crop plants to meet such requirements. Its high nutritional value is comparable to soy. Moreover, almost the whole body of the hemp plant has a wide array of utility: industrial production of food, fiber, and construction materials. In view of environmental sustainability, hemp requires less pesticides or water in cultivation compared to cotton, a representative fiber plant. This short review investigates hemp’s sustainability as a plant as well as its utility value as a highly nutritional material in the food industry. Recent application research of hemp protein in food processing includes plant milk, emulsifiers, fortification of gluten-free bread, plant-based meat production, as well as membrane formation. These studies have revealed distinctive properties of hemp protein, especially in relation to disulfide (S-S)/sulfhydryl (-SH)-mediated interactions with protein from other sources. While its cultivation area and industrial use were limited for a while over confusion with marijuana, the market for industrial hemp is growing rapidly because it has been highly reevaluated in multiple areas of industry. Conclusively, with its sustainability as a plant as well as its distinctive useful property of the seed protein, hemp has promising value in the development of new foods.

## 1. Introduction

The industrial revolution has rapidly enhanced the standard of living. However, large-scale factory production as well as modernization of agriculture have caused serious environmental problems which now threaten the existence of human beings [1]. Global warming has raised water deficiency problems [2]. Long-term use of pesticides in agriculture brought severe environmental pollution [3]. Moreover, the explosive growth of the world population and increasing number of meat-eaters not only imbalanced the supply/demand of livestock meat but also accelerated the food crisis [4]. Thus, in 2015, the United Nations adopted the 17 Sustainable Development Goals and quoted that development must balance social, economic, and environmental sustainability. Moreover, the prevailing COVID-19 situation and the Russia–Ukraine conflict remind us of the importance of food security [5,6,7]. Now, the food industry seeks protein sources from plants rather than from livestock animals as the plant cultivation poses less impact on the environment [8,9]. Hemp and its industrial products look to be one of the prospective keys in developing sustainable and resilient food systems [10]. In the viewpoint of cultivation, hemp has a short cropping period [11] and needs less pesticide or water compared to cotton [12], a representative fiber material plant. Moreover, the whole body of the hemp plant—its leaves, stalks, roots, and seeds—are utilizable without waste [13]. Furthermore, hemp seeds are comparable with soybeans in terms of nutrition [14].

Hemp has historically close relations with humans. Industrial hemp has long been used in various civilizations and religions since ancient times to the present day [15]. In Japan, a Shinto priest uses cannabis leaves to purge and bless the worshippers in a shrine where thick ceremonial hemp ropes are displayed. In the practical viewpoint, hemp played outstanding roles, especially in the Age of Exploration, as an essential material to make durable canvas and ropes for sailing ships. Later, in the World Wars era, the United States made full use of hemp as a reliable material to cover a shortfall of essential fiber products such as military uniforms and shoelaces, as well as parachutes while fully engaged in combat [16]. However, for a period of time, confusion with marijuana has limited the cultivation area and industrial use of hemp. Now, due to its sustainable growth characteristics as well as its versatile industrial usability, hemp is reevaluated as a promising crop in the era of SDGs [11]. The stalk is for fiber production, the leaves/roots for medicine, and seeds for oil until nothing is left. Moreover, its high nutritional value is comparable to soy and its unique characteristics in food processing appear to be high-profile in the food industry. Moreover, with its gluten content as low as 4.78 ppm, it is attracting attention as a gluten-free (<20 ppm) food material [17]. Oil meal, a residual material of hemp seed after expression of hemp oil, is a useful protein-rich material [18]. Recent application of hemp protein in food processing reveals its unique properties. Especially, in recent studies, its cysteine-rich amino acid composition and high sulfhydryl (-SH)/disulfide (S-S) ratio offer a glimpse of its distinctive features useful in food processing [19]. This short review verifies the usefulness of hemp focusing on its sustainability as a plant as well as its usability as a versatile food material. The authors also briefly introduce the historical relationship between human and hemp.

## 2. A Brief History of Hemp Cultivation in the World

A recent study based on genome-wide phylogeography supports the leading hypothesis that *Cannabis sativa* was first domesticated in East Asia in the early Neolithic era [20]. Ren et al. [20] demonstrated that all current hemp and drug cultivars diverged from an ancestral gene pool currently represented by feral plants and landraces in China. *Cannabis sativa*, or “useful hemp” in Latin, is categorized into non-drug type hemp and drug type marijuana depending on the content of psychoactive compound delta-9-tetrahydrocannabinol (THC) [21]. Industrial hemp contains only about 0.3–1.5% of THC, whereas marijuana contains 5–10% or more of THC. Hemp contains cannabidiol (CBD), a non-intoxicating phytocannabinoid, which has high medicinal potential in some conditions, such as difficult-to-treat seizures in children and adults [22].

The history of hemp and human society is long and complex—from being an essential commodity fiber crop in the Age of Discovery to its widespread prohibition under the umbrella of marijuana in the 20th century. The oldest known fossil pollen record compatible with cannabis was found in rocks 19.6 million years old (Early Miocene) from the north-eastern Tibetan Plateau (presently China), which has been proposed as the center of origin of cannabis [23]. There, hemp has been grown for 4000 and 6000 years for the production of textiles and fiber. However, in 1985, China banned the production of hemp after it ratified the UN Convention on Psychotropic Substances [24]. Hemp production became legal again in 2010, and now the Chinese government encourages the textile industry, such as the Youngor Group, to manufacture hemp fiber products [25]. Although official data for China’s hemp cultivation and production are unavailable, industry estimated China’s hemp planted area at around 66,700 hectares (165,000 acres) in 2019. China’s hemp market value was also estimated at USD 1.7 billion in 2017 [24].

Hemp is also grown across Europe. The cultivation area in the EU has increased significantly from 19,970 to 34,960 ha between 2015 and 2019. The production of hemp increased from 94,120 to 152,820 tons in the same period. France is the leading hemp cultivator, making up for more than 70% of the overall EU production, followed by the Netherlands (10%) and Austria (4%) [26]. In the UK, in 1533, King Henry VIII mandated every farmer to cultivate hemp, a useful source of tough fiber to produce naval equipment such as rope, canvas, and sails to protect the land surrounded by the ocean [27]. However, in 1961, the Single Convention on Narcotic Drugs banned all forms of cannabis in the US and in Western Europe (except France) because of the confusion between hemp and marijuana. Note, as an aside, that Boris Johnson mentioned climate protesters as “hemp-smelling bivouacs” [28].

Hemp has played critical roles in American history. The Declaration of Independence was drafted on hemp paper. George Washington, the first president of the US, was to be found exhorting his head gardener to: “Make the most of the Indian hemp seed…and sow it everywhere” [29]. Federal restrictions on use or sale of cannabis first occurred with the passage of the Marihuana Tax Act of 1937 (“the Act”) following the repeal of the National Prohibition Act that prohibited the production, importation, transportation, and sale of alcohol from 1920 to 1933. The Marihuana Tax Act imposed registration requirements and a tax on growers, sellers, and buyers of marijuana [30]. However, during wartime, the US government relied on hemp to make twine, tarred cordage, nets, shoelaces, carpets, and parachutes. After World War II, hemp has been cultivated in very small amounts and eventually found itself “unjustly imprisoned” [16] under the negative influence of its sister plant, marijuana. In 2018, the Federal Farm Bill has legalized hemp as an agricultural commodity and removed it from the controlled substances list [31]. Now, as of 2020, the US seems to be the world’s largest producer of industrial hemp with a licensed area of 465,787 acres [32].

The world market of industrial hemp was estimated to be USD 4.13 billion in 2021 and is expected to grow by a 16.8% compound annual growth rate (CAGR) between 2022 and 2030 [33]. Growing demand for industrial hemp from a wide variety of application industries drives the market [34]. While the global market is undergoing limited growth under the influence of the COVID-19 pandemic, it is expected to grow at a faster rate with the high recovery rate of global economies [33].

## 3. Reevaluation of Hemp for Its Sustainability

Hemp has received a lot of attention because of its multipurpose usability, short production cycle, and low capital demand in cultivation, possibility as a carbon-negative material [11]. Here, recent examples are introduced to show how hemp is excellent as SDG-applicable materials.

### 3.1. Cellulose Gap: Applications in the Textile Industry

During the Age of Exploration, hemp was a critical crop in Europe for the production of strong fiber suitable for canvas and ropes for sailing vessels. However, around the 18th century, cotton had become popular and had taken over the role as the principal fiber plant. Cotton fiber has a soft and comforting touch because cellulose of high crystallinity and purity compose the cell wall [35]. Now, the “cellulose gap”, an excessively high demand for natural fiber not being met by cotton production, encourages textile manufacturers to seek substitutes for cotton [12,36]. Moreover, while cotton is grown on only 2.4% of the cropland in the world, its cultivation uses 11% of the world’s pesticides [37]. Industrial hemp is one of the most relevant substitutes for cotton because it requires less pesticides and water in cultivation [12]. Duque Schumacher et al. [38] has demonstrated that hemp requires only one-third of the land area occupied by cotton to produce the same amount of fiber while cotton needs 2.5 times more water than hemp per unit cultivation area. Duque Schumacher et al. [38] concluded that in terms of agricultural activities for fiber production, hemp costs only one-twelfth of the cost for cotton. Especially, the ‘Henola’ variety has a shorter growing period as well as higher seed productivity, and thus looks preferable for introduction and cultivation [39].

Meanwhile, hemp fiber is known as coarse and stiff. It also has poor spinnability in the ring spinning system [40]. The blended use of cotton/hemp fibers for denim fabrics improved the thermo-physiological comfort as well as the soft-feeling nature. Moreover, higher air ventilation, higher water-absorbing property, and faster drying behavior were observed in the mixed fabrics compared to a pure cotton fabric [41].

### 3.2. Hempcrete

Hemp concrete or “hempcrete” is made of lime, water, and hemp shives, a by-product of fiber processing from hemp stalk. It is a bio-composite material used as an alternative to concrete for construction and insulation. Hempcrete is “carbon-negative” or “better-than-zero-carbon” because hemp plant absorbs more carbon from the air during growth than it yields during its production. Moreover, it continues to absorb carbon after being employed in construction, storing more carbon over the building’s lifetime than was emitted during construction [42]. In 2021, Pierre Chevet Sports Centre was built in France as the first public building using hemp concrete as the main construction material [43]. Moreover, preliminary 3D-printing research shows that hempcrete is printable with a density as low as 660 kg/m^3^, with adequate buildability and compressive strength for printing individual walls [44]. Such technical progress will widen the uses of hemp and the possibility to alleviate the negative impact of the construction industry on the environment.

### 3.3. Biofuels

Human activities in industry and transportation have resulted in an extensive use of fossil fuels, negatively affecting the environment causing climate change, as well as global warming. Bioenergy is a green alternative for diverse energy needs. Thus, novel technology realizing effective conversion processes must be developed to enhance affordable biofuel production [45]. Marrot et al. [46] investigated the influence of the thermochemical conversion processing parameters on energy production as well as electrical conductivity. Two distinct scenarios of hemp biomass valorization were proposed, depending mainly on the selective pyrolysis temperature. Hemp biochar carbonized at 400–600 °C was classified as a lignocellulosic material with good potential for solid biofuel applications due to its high heating value. In contrast, hemp biochar carbonized at 800–1000 °C developed a graphite-like microstructure and displayed interesting electrical conductivity, opening doors for its use in electrical purposes.

### 3.4. Bioplastics

Petroleum-based plastics cause many environmental problems, such as marine pollution, human health problems, and greenhouse gas emissions. On the other hand, bioplastics are drawing attention as alternatives to conventional ones [47]. Short hemp fiber is used to recycle polypropylene from textile wastes into wood plastic composites [48]. Cast hemp paper is laminated with bio-based plastics for sustainable packaging [49]. Hydrothermal and mechanically generated hemp hurd nanofibers are utilized for sustainable barrier coatings/films [50]. Hemp-based fiber is being applied even to the interior of high-end vehicles [51]. Hemp-reinforced natural fibers have been used in the interior components of the Mercedes-Benz E-Class. A significant portion of the Lotus ECO Elise’s body panels are manufactured using hemp fiber-reinforced polyester composite.

## 4. Nutrition

### 4.1. Overview

**Figure 1:**
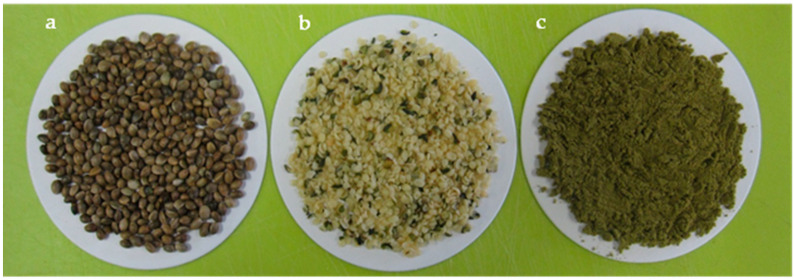
Whole (**a**) and dehulled hemp seed (**b**). Hemp flour (**c**).

The nutritional value of hemp is attracting attention [14]. Hemp seed is composed of a white kernel and brown hull (Figure 1). The kernel is rich in protein, unsaturated fatty acid, and dietary fiber [52]. Hemp shares a unique high-protein, low-carbohydrate nutritional composition with soybean, distinctively different from other representative food materials such as rice and wheat, the protein/carbohydrate (%, *w*/*w*) content of which is 7.17/77.55 [53] and 10.6/73.2 [54], respectively. Hemp and soy are also rich in dietary fiber and unsaturated fatty acids (Table 1). Hemp oil is popular and the oil meal, a by-product of oil processing, is utilized in many protein-rich foods as well as animal feeds [55].

### 4.2. Protein

#### 4.2.1. Composition of Hemp Protein (Globulin, Albumin, and Others)

The major protein in hemp kernel is edestin, accounting for around 70% of hemp protein. Edestin is a hexamer of identical subunits and belongs to the globulin family [57]. Each subunit consists of the acidic (−34 kDa) and the basic (18–20 kDa) chains [58]. An original single protein is cleaved into these two chains post-translationally at the Asn-Gly site [57]. An edestin subunit has five cysteine residues, two of which form a single intermolecular disulfide bond between basic and acidic subunits. The acidic subunit has one intramolecular disulfide bond. The remaining cysteine has a free sulfhydryl (SH) group. Edestin is less soluble in water or buffer with neutral or acidic pH, but soluble in a basic buffer [59]. As solubility in water and content of free SHs increases by sonication or pH adjustment of the edestin solution, control of the protein structure/function may be possible in food processing [60]. Despite its less solubility, edestin is known for its high digestibility [61,62].

The second major hemp protein is albumin, which has fewer disulfide bonds compared to edestin (globulin), thus having a flexible structure with higher protein solubility and foaming capacity [59]. Another noteworthy protein is rich in Met and Cys, and 20 mole % of the total amino acids contain sulfur [63]. It consists of two subunits made of 27 and 61 amino acid residues, respectively, which are held together by two intermolecular disulfide bonds [63].

#### 4.2.2. Extractability/Solubility

Recently, reverse micelles (RMs) technology has been applied for protein extraction as it is convenient and cost-effective [64]. Protein extraction by RMs is achieved via two steps: “forward and backward” extractions. In the forward extraction, soluble proteins are encapsulated in the inner aqueous core of RMs, which are then recovered in the backward extraction by disrupting the RMs. Protein extraction from hemp flour using RMs did not require prior defatting in terms of extraction performance [65]. Hemp protein isolates obtained from non-defatted and defatted hemp flour shared similarities in proximate composition, subunit structure, as well as principal properties of protein including solubility, isoelectric point, emulsifying/foaming abilities, and thermal stability. Meanwhile, protein from non-defatted flour exhibited higher β-sheet content and surface hydrophobicity. Furthermore, higher least gelling concentration and higher gel strength were observed in the gel formed by protein from non-defatted hemp flour [65].

#### 4.2.3. Digestibility/Allergenicity

In vitro digestion tests demonstrated that hemp protein has a high degree of digestibility [61]. Moreover, most hemp allergens, such as the major thaumatin-like protein and lipid transfer protein, were eliminated in the protein-isolation or digestion processes. Mamone et al. [61] concluded that hemp protein is usable as an ingredient for hypoallergenic foods.

#### 4.2.4. Disulfide Structure

Hemp protein has a unique feature in view of the disulfide structure. It shows much higher free sulfhydryl content than soy protein [66]. The cysteine content of hemp protein is 1.6–1.4 g cysteine/100 g protein [32,67], higher than pea (1.0 g cysteine/100 g protein) and broadbean or soybean (1.1–1.3 g cysteine/100 g protein) [68]. Moreover, at pH 8, while the free sulfhydryl content of soy protein is 1.6 × 10^6^ mol/g protein, the counterpart of hemp is as high as 3.9 × 10^6^ mol/g protein [66]. Tang et al. [66] discussed that the poor functional properties of hemp protein seems mostly ascribed to the formation of intermolecular disulfide bonds between individual proteins and subsequent aggregation at neutral or acidic pH, because it has a high free sulfhydryl content. Meanwhile, exchange reactions between disulfide (S-S) and free SH play critical roles in the cereal biology [69] as well as food processing [70,71]. In the preparation of microparticles made from soy protein isolate and egg white, the egg white protein serves as a free sulfhydryl “donor” to accelerate intermolecular S-S linkage with the soy protein [72]. Thus, hemp protein with a high free SH content should have unexplored unique processing characteristics. Heat-dependent formation of the envelope made of hemp and soy protein is just a glimpse of this attribute [73,74].

## 5. Applications of Hemp Protein to the Food Processing Industry

### 5.1. Hemp Milk

Increasing demand for plant-based milk has enlarged its industry due to lactose intolerance, cow’s milk allergy, and vegan lifestyles [75]. The principal predictor of dairy consumption in the acceptance of dairy products was “Nice”, relative to the other 3Ns, that are, Natural, Necessary, and Normal. “Taste” was the most relevant keyword in consumers’ selection of dairy products [76]. Hemp milk, with its highly nutritional value and low allergenicity, looks to be an attractive alternative to dairy, soy, and nut milks. Comparison of milk products in the market reveals hemp milk as a better source of minerals than other dairy and plant milks. Hemp milk does not taste that different but has a “nuttier” flavor in comparison to soy or rice milk [77]. High pressure homogenization following pH adjustment realized non-thermally processed hemp milk, which is remarkably stable, showing negligible phase separation in storage for 3 days at 4 °C [78].

### 5.2. Emulsifier

**Figure 2:**
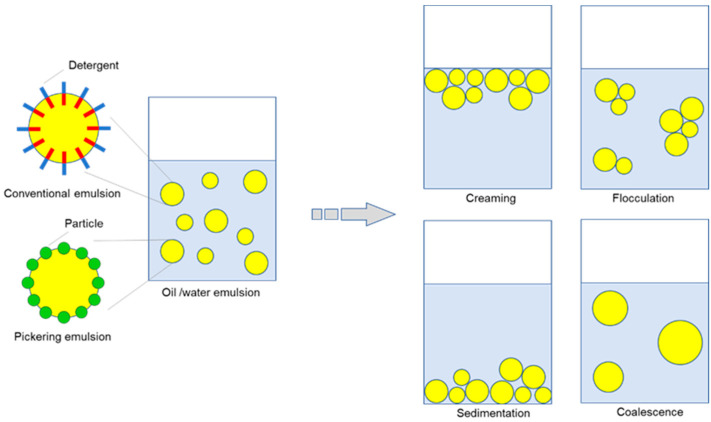
Formation of oil/water emulsion and its four major destabilization patterns.

Stabilization of emulsion-based colloidal structures by food ingredients has attracted attention in the food and pharmaceutical, as well as cosmetic, industries [79]. In the Pickering emulsion [80,81], solid particles are adsorbed onto the interface between the two distinct phases, such as oil and water, and inhibit emulsion droplets from coalescence (Figure 2). Stabilizing the oil/water boundary by plant-based materials such as proteins and organelles is useful in exploring emulsion systems in developing new foods [82]. Examples include egg-free mayonnaise [83], barley-based non-dairy milk [84], probiotic encapsulation [85], as well as additive-free, gluten-free rice bread [86]. However, these bio-based emulsions tend to be unstable due to gravity-induced separation (creaming/sedimentation), flocculation, and coalescence (Figure 2).

Hemp protein is considered a novel emulsifier in food systems. Dapčević-Hadnađev et al. [87] compared the behavior of two types of hemp proteins in the sunflower oil-in-water emulsions. Salt extracted “micellar” hemp protein (HMI) with a less-denatured structure exhibited higher solubility as well as slightly higher surface/interfacial activity than alkali-extracted and isoelectric-precipitated hemp (AIH) protein. HMI formed emulsions composed of relatively small droplets with enough static repulsion between droplets. Individual droplets were covered by protein film. However, low viscosity of HMI-stabilized emulsions facilitated fast droplet movement and eventually led to increased creaming and coalescence at lower protein concentrations (0.25–0.75% *w*/*w*). Meanwhile, AIH exposed hydrophobic sites as well as sulfhydryl groups due to pH-induced unfolding of protein structure. In AIH-stabilized emulsions, bridging flocculation occurred during emulsification by the formation of protein-connected droplet aggregates. However, interestingly, emulsions stabilized with 1.5% (*w*/*w*) AIH showed creaming and coalescence stability much better than emulsions with lower AIH concentrations. Formation of a weak transient network of floccules as well as higher continuous phase viscosity suppressed the movement of the droplets, resulting in the improvement of the emulsion’s stability.

Feng et al. [88] sought to make complexes of pectin and hemp protein through electrostatic force. Monodisperse features of hemp protein in the aqueous phase improved by the increase of surface charges as well as the blockage of free SH groups. The presence of pectin inhibited the coalescence and provided substantial physical strength to ensure stability during the storage test. On the other hand, Li et al. [89] sought to stabilize hemp protein-mediated oil/water emulsion by high-intensity ultrasonic treatment. The emulsifying properties were improved, showing a more uniform particle distribution with small and well-dispersed particles. With its unique behavioral attribute in the oil/water interface, hemp protein is expected as a promising competitor against zein [90], well-used in the Pickering-based food systems.

### 5.3. Gluten-free Bread

Due to the world-wide prevalence of celiac disease and wheat allergy, demand for gluten-free foods, especially for bread, is increasing [73]. However, most gluten-free breads are starch-based and thus generally low-protein and high-carbohydrate. Therefore, researchers are trying to improve the nutritional value of gluten-free breads by fortification of nutrients [91]. Hemp is one of the ideal food materials because of its high protein content, low content of saturated fats, and high content of unsaturated fatty acids such as ω3 and ω6 [92].

Addition of hemp protein concentrate significantly improved the nutritional value of the starch-based gluten-free bread [93]. It also changed the rheological characteristics of the gluten-free dough and reinforced the structure. Moreover, sensory acceptance on color and flavor was confirmed for the bread. Limited amylopectin recrystallization as well as limited hardening of the crumb were observed during storage.

Evaluation of the effect of hemp seed flour addition and following sourdough fermentation on the aroma of gluten-free bread was conducted [94]. By a metabolomic approach, production of volatile organic compounds of flavoring and health benefits were identified. Among them, 1-heptanol is a food flavor generally used to confer a musty, pungent leafy and green nuance [95]. The odor of 2-heptenal is described as a pleasant almond flavor. 2-pentanone-3-hydroxy has a caramel-sweet, buttery, and hay-like aroma [96]. On the other hand, hexanoic and octanoic acids with unpleasing odor were also identified. Therefore, strategic adjustment of the dough composition as well as baking conditions are the challenges for future studies for a balanced flavoring of the bread.

We are so accustomed to the fresh aroma of wheat bread. Thus, in developing bread without using wheat flour, exquisite formation of its aroma is critical for it to be accepted by consumers. Nissen et al. [94] proposed the utilization of hemp flour as a vehicle to carry the flavor and bioactive compounds in bakery products. Volatilomics appears to be a useful technical tool in designing the organoleptic characteristics of gluten-free breads.

### 5.4. Hemp Meat

Demand for high quality vegan meat made of plant materials is increasing based on the shortage of animal stock due to the global increase of human population, as well as in view of animal welfare [97,98,99].

#### 5.4.1. Hemp as a Nutrient Feed Stuff

Hemp cake, a residual material of oil expression from the hemp seed, has long been utilized for livestock feeds [100,101]. It is a highly nutritive as well as sustainable feed stuff for cows [102], quails [103], cockerels [104], pigs [105], and broilers [106]. Now, research is in progress to utilize hemp protein “directly” as a material for plant meat.

#### 5.4.2. Vegan Meat Made of Hemp/Soy Proteins

The commercially available vegan meat products, represented by high moisture meat analogues (HMMAs), are made mostly from soy. In the production of HMMA, protein-rich materials, such as soy protein isolate, are subjected to a twin screw co-rotating extruder. Thermomechanical stresses are applied to the material at a high-water content (>40% *w*/*w*) followed by forcing through a cooling die [107]. Soy-based HMMAs have a desirable chewy meat-lite texture. However, cultivating soy in colder climates such as in northern Europe is challenging. Thus, Zahari et al. [108] sought to investigate whether and to what extent soy protein isolate could be replaced by hemp protein concentrate in the production of HMMAs. A rapid visco analyzer (RVA) and differential scanning calorimeter (DSC) were used to investigate pasting features and melting temperature of the raw materials. They found that hemp protein absorbed less water and requested higher temperature for denaturation compared to soy protein. However, replacement of soy protein with hemp protein was possible up to 60% to yield layered and fibrous meat-like extruded products. Based on the DSC and RVA results, a higher cooking temperature and longer retention time are recommended for the extrusion of hemp/soy meat, as hemp protein needs a higher temperature for denaturation. Furthermore, to develop a more laminar fiber structure, the interior structure of the extruder should be equipped with more complicated kneading elements such as screws to hold the material longer in the extruder. Zahari et al. [108] concluded that while future studies are needed to optimize the condition of the extrusion process and the formulation matrix, it is possible to substitute soy protein with hemp protein without sacrificing the quality in meat analogue formulation.

#### 5.4.3. “Meaty” Hemp Meat: Anisotropy and Fibrousness

To develop plant-based meat with a realistic “meaty” texture, anisotropy and fibrousness are among the most critical factors [109,110]. Anisotropy is the property of a material expressing different behaviors depending on the directions from which the external pressure is applied [111]. Meat structure is highly directional. On the molecular scale, actin and myosin form well-ordered and parallel arrays of filaments. On the macroscale, those filaments form into the muscle fiber bundles [112].

During a high-moisture extrusion of plant protein-based materials, the dynamics of protein aggregation and phase separation are the keys for the formation of meaty fibrous structures. The fibrousness is expressed during migration from the die to the cooling zone through a “sub-layer transformation” cross-linking [113]. The desired anisotropic structure of plant-based meat analogues has been accomplished by extrusion at high water content (>40%) and at elevated temperatures (>100 °C) followed by passing through a cooling die which prevents expansion of the matrix at the ejection from the extruder [114]. Interestingly, there are two distinct hypothetical mechanisms to explain how the anisotropic structure is made in the extruded plant meat. One is the “cross-linking” mechanism in which the anisotropic structure is explained as being formed by the alignment of protein and the subsequent stabilization at the molecular level. Protein molecules are unfolded and align along the direction of flow, followed by stabilization of the aligned proteins by way of interactions between/among proteins newly developed by disulfide or hydrophobic interactions.

The other is the “multiphase” mechanism in which formation of the anisotropic structure is explained due to the existence of multiphase systems [114]. Thermodynamic immiscibility of the biopolymers involved triggers the occurrence of phase separation in the extrusion process. The dispersed phase is deformed in the extruder die, then directed along the flow. The subsequent cooling process solidifies the material resulting in the anisotropic structures of the plant-meat products. In the case of extrudates using soy protein isolate solely as a protein source, the formation of anisotropic structures are derived from a multiphase system. Cryo-imaging and X-ray analysis of the extrudates revealed a water-rich dispersed phase surrounded by a continuous protein-rich phase with less moisture. Meanwhile, significant changes of protein–protein interactions were not observed [114]. Thus, in this model system, multiphase systems rather than cross-linking of proteins seemed to be the primary factor of the anisotropic structure.

On the other hand, during high-moisture extrusion processing of meat analogues made of pea protein and fatty acids, protein–protein interactions played key roles in the product structure [115]. Micromorphology analysis demonstrated that formation of anisotropic fibrous structures in the cooling die was disturbed by the coalescence of fatty acids of an unsaturated type, such as oleic and linoleic acids. Meanwhile, saturated stearic acid dispersed uniformly in the protein matrix, facilitating formation of disulfide bonds and promoting the generation of anisotropic fibrous structures along the extrusion direction [115]. Moreover, in the case of plant meat made by high-moisture extrusion processing of pea protein, amylopectin, and stearic acid, its anisotropic fibrous structures have been explained by the “anchor orientation and flexible cross-linking” mechanism [116]. In the cooling zone, stearic acid played the role of anchors, preventing the unfolded protein structure from refolding. In contrast, amylopectin facilitated the rearrangement, disulfide formation, and polymerization of the protein molecules. Thus, amylopectin and stearic acid synergistically mitigated the interaction between proteins. These aggregates with loose and flexible structures aligned along the extrusion direction and successfully formed anisotropic and fibrous structures in the extruded products.

Nasrollahzadeh et al. [67] compared the structure of plant meat made of hemp protein with the counterpart made of pea proteins. The proteins were respectively mixed with maize starch and were subjected to high moisture extrusion. The extruded pea meat was soft and isotropic while hemp meat was hard and anisotropic. The protein structure was investigated using SDS-PAGE in the presence or absence of a reductant, dithiothreitol (DTT). In the case of the hemp meat sample, some protein bands derived from edestin appeared only in the presence of DTT. In contrast, less-intense protein bands of pea meat appeared regardless of the presence or absence of the reductant. The results demonstrate a higher contribution of disulfide cross-linking in the polymerization of hemp protein during the extrusion process than in the case of pea protein. As mentioned above, hemp protein contains more cysteine (1.6–1.4 g cysteine/100 g protein) than pea protein (1.0 g of cysteine/100 g protein). Thus, in the case of hemp meat, protein–protein interactions played critical roles in the formation of anisotropy and fibrous-like mesoscale structures. More recent studies support the view by demonstrating that addition of cysteine controls the texture of plant meat. Addition of cysteine changed both the physical and chemical properties of extrudates made from soy protein isolate and wheat gluten [117]. The SH-containing amino acid promoted the fiber structure formation and affected the degree of texturization and rheological properties, as well as microstructures of the extrudates by rearranging the disulfide-mediated cross-linking among protein molecules [117]. Meanwhile, addition of L-cysteine or L-ascorbic acid on the material of the pea protein/wheat gluten blend altered the fibrousness and the mechanical properties of the meat analogue obtained by the high-temperature shear cell [118]. Cysteine accelerated protein polymerization through the disulfide–sulfhydryl exchange reactions in the heating process, yielding a continuous protein network upon cooling.

Conclusively, the alternative mechanism of the generation of fibrousness and anisotropy in the plant meat, whether cross-linking or multiphase, depends on the protein species, extraction process of the protein, and the subsidiary materials such as starches and fatty acids. In the case of hemp protein, its high content of free sulfhydryl groups is expected to produce the unique “meaty” texture of the products.

#### 5.4.4. Membrane Formation

**Figure 3:**
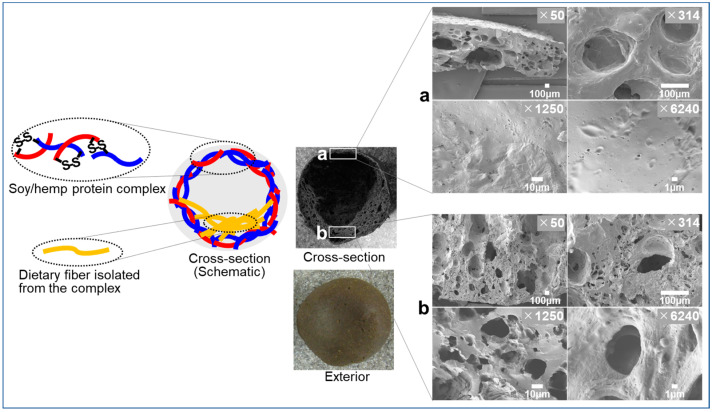
A “roly-poly” wiggle ball made from soy/hemp protein complex. (**a**), protein-rich outer shell; (**b**), fiber-rich inner bottom part. Reprinted/adapted with permission from Ref. [74]. Copyright 2022, NARO.

Recently, it was reported that a dough made of egg white and soy protein isolate was baked into a high-protein, low-carbohydrate bread [73]. Egg white protein and soy protein formed heat-dependent elastic air-cells which work to confine enlarging gas in the baking process. Intermolecular S-S bonds contribute to the formation of the cell membranes in which egg white protein works as a donor of SHs to soy proteins [72,73]. Similarly, a dough made of hemp protein/soy protein is baked into a hollow ball (Figure 3) [74].

The outer shell is made of the mixture of soy/hemp proteins which are linked with intermolecular disulfide bonds. Dietary fiber, isolated from the protein complex, is located in the bottom of the ball. Thus, the ball has a low center of gravity and behaves like a “roly-poly” wiggle ball. In this case, hemp protein, rich in free SHs [63,66], is considered to work as a SHs donor, as egg white protein worked as a donor of free SHs in the bread made of egg white/soy protein isolate [73]. Electron microscopic observation of the protein-rich outer shell (Figure 3a) and fiber-rich inner bottom part (Figure 3b) reveals distinct structures. The former has a dense structure while the latter has a reticulated structure with small void spaces. Contribution of the unique structure to the ball’s texture is being investigated by sensory evaluation in our lab. Conclusively, application of hemp protein on food processing will broaden the variation of the food products.

## 6. Comparative Summary of Hemp, Cotton, and Soybean

**Figure 4:**
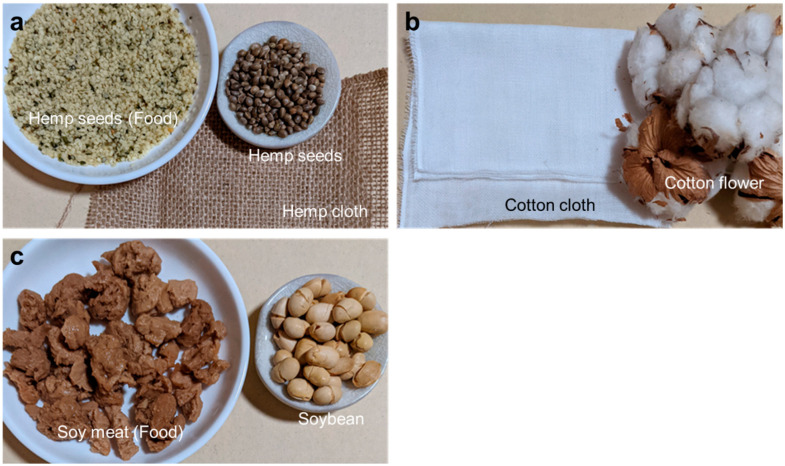
Examples of the industrial application of hemp (**a**), cotton (**b**), and soybean (**c**).

The industrial utility of hemp, cotton, and soybean is outlined in Figure 4 and Table 2. Hemp is usable as both a fiber and food material (Figure 4a). Cotton is used mostly for fiber production, while soybean for food processing (Figure 4b,c). As all seeds are available for oil expression, and the residues are utilized as a highly nutritional food material (hemp and soybean) or for fiber production (hemp and cotton), they are all deemed sustainable. However, as hemp is more sustainable than cotton or soybean in view of cultivation and utility, ++ was marked only for hemp in the sustainability row (Table 2).

## 7. Conclusions

Hemp is a sustainable plant requiring less water or pesticides in cultivation compared to cotton. It has a short growth period and almost its whole plant body has versatile utility value. Hemp seeds are high-protein, low-carbohydrate, and rich in dietary fiber and unsaturated fatty acids. After expression of oil from the seeds, the residual mass is a useful protein-rich material for food processing. Moreover, hemp seed protein has distinctive characteristics suitable for developing new foods such as an emulsifier, plant-based meat, and gas-retaining membrane. The cysteine-rich protein feature realizes unique disulfide-mediated interactions with protein from other sources and is thus expected to facilitate development of new food materials. Meanwhile, hemp protein is reported to be less soluble, and a higher temperature is needed for processing compared to other plant protein. Therefore, suitable reaction conditions should be investigated for future application in the food industry. Further scientific understanding will facilitate expanded use of this less-investigated protein compared to soy protein. Conclusively, hemp is a suitable plant with versatile utility in this SDGs era. Hemp seeds and the protein are expected to be promising food materials in the food industry.

## Figures and Tables

**Table 1:** Comparison of nutritional components between hemp seed and soybean.

	Whole Hemp Seed	Soybean
Protein	23.54	34.96
Carbohydrate	30.89	31.6
*Sugar*	2.01	3.99
*Dietary fiber*	28.88	27.61
Fat	32.28	22.19
*Saturated fat*/*Total fat* (%)	11.32%	13.77%
*Unsaturated fat*/*Total fat* (%)	89.06%	86.23%

This table was made using data from Teleszko et al. [39] and Kan et al. [56].

**Table 2:** Comparative summary of hemp, cotton, and soybean in view of sustainability as a plant, as well as industrial applications.

	Hemp	Cotton	Soybean
Sustainability	++	+	+
Fiber/Textile use	+	+	-
Nutritional use	+	-	+
Distinctive Food processing feature of the protein	SH donor	-	SH recipient

++, Very applicable; +, applicable; -, not applicable.

## Data Availability

Data is contained within the article.
